# A Novel Centralized Range-Free Static Node Localization Algorithm with Memetic Algorithm and Lévy Flight

**DOI:** 10.3390/s19143242

**Published:** 2019-07-23

**Authors:** Jin Yang, Yongming Cai, Deyu Tang, Zhen Liu

**Affiliations:** 1 School of Medical Information and Engineering, Guangdong Pharmaceutical University, Guangzhou 510006, China; 2 Guangdong Province Precise Medicine and Big Data Engineering Technology Research Center for Traditional Chinese Medicine, Guangzhou 510006, China

**Keywords:** wireless sensor network, node localization, quantum-behaved particle swarm optimization, Lévy flight, memetic algorithm

## Abstract

Node localization, which is formulated as an unconstrained NP-hard optimization problem, is considered as one of the most significant issues of wireless sensor networks (WSNs). Recently, many swarm intelligent algorithms (SIAs) were applied to solve this problem. This study aimed to determine node location with high precision by SIA and presented a new localization algorithm named LMQPDV-hop. In LMQPDV-hop, an improved DV-Hop was employed as an underground mechanism to gather the estimation distance, in which the average hop distance was modified by a defined weight to reduce the distance errors among nodes. Furthermore, an efficient quantum-behaved particle swarm optimization algorithm (QPSO), named LMQPSO, was developed to find the best coordinates of unknown nodes. In LMQPSO, the memetic algorithm (MA) and Lévy flight were introduced into QPSO to enhance the global searching ability and a new fast local search rule was designed to speed up the convergence. Extensive simulations were conducted on different WSN deployment scenarios to evaluate the performance of the new algorithm and the results show that the new algorithm can effectively improve position precision.

## 1. Introduction

The sensed data gathered by sensor nodes is only useful when the location information of these sensors is known in a variety of emerging applications of WSNs [1]. However, most nodes in WSNs are deployed in an ad-hoc manner without any prior knowledge of their location information. Therefore, how to determine the location of an unknown node is an essential issue. Equipping each node with a global positioning system (GPS) receiver seems to be a simple and effective solution, but this scheme is unrealistic due to its high cost and energy consumption, especially for large-scale WSNs. Additionally, the poor performance of GPS inside an indoor environment also makes GPS an impractical scheme. The more reasonable solution is to assume that only a small number of nodes (called anchors) own their position, while the others (called unknown nodes) can estimate their position according to the anchors.

In recent years, many localization techniques have been proposed, and a detailed survey of the relevant literature is available in [2]. Generally, existing schemes can be categorized as range-based and range-free. The range-based schemes, such as RSSI [3], AOA [4] and TDOA [5], require absolute point-to-point information of distance or angle between nodes for localization; these schemes have a higher localization accuracy but they require additional expensive hardware to measure the information of distance or orientation. Moreover, these schemes can be affected easily by multipath and noise. Therefore, the range-based ones are impractical for resource-limited WSNs.

In contrast, the range-free schemes do not require the absolute information of distance or angle or other physical measurements among nodes for localization. They only need the network connectivity information. The unknown nodes can estimate their position according to the connectivity information and the anchors’ position. Thus, these schemes do not require any additional hardware and provide a cost-effective solution. DV-Hop [6], Centroid [7], APIT [8] and Grid_Scan [9] are typical range-free examples. However, the ranged-free schemes always have poor positioning accuracy. Nevertheless, their cost-effectiveness and simplicity motivate researchers to improve their localization performance.

In the swarm intelligent algorithm-(SIA) based solutions, the localization problem is formulated as an *NP*-hard optimization problem, and many optimization algorithms are applied to solve it. The general optimization model (i.e., the fitness function) can be summarized as follows.

(1) $$\mathrm{Min}\;f_{i}=\sum_{k=1}^{n}\omega_{k}\times {\left(d_{ik}-{\bar{d}}_{ik}\right)}^{\alpha }\;0\leq \omega_{k}\leq 1\;\mathrm{and}\;\sum_{k=1}^{n}\omega_{k}=1$$

where i is the ith unknown node, n is the number of anchors. Here, an unknown node can be treated as an anchor if its position has been estimated.$\omega_{k}$ is a weight factor which is used to indicate the importance of each anchor. α is the exponent factor which is used to adjust the effect of the measured distance error on the result. ${\bar{d}}_{ik}$ is the estimated distance between the ith unknown node and kth anchor. Different localization algorithms require different calculation methods to estimate ${\bar{d}}_{ik}$, for example, RSSI-based methods and a method based on the number of hops. $d_{ik}$ is the actual distance between the ith unknown node and the *kth* anchor, which can be calculated as

(2) $$d_{ik}=\sqrt{{\left(x-{\overset{⌢}{x}}_{k}\right)}^{2}+{\left(y-{\overset{⌢}{y}}_{k}\right)}^{2}}$$

where (x,y) is the coordinate of the ith unknown node, and $\left({\overset{⌢}{x}}_{k},{\overset{⌢}{y}}_{k}\right)$ $\left({\hat{x}}_{k},{\hat{y}}_{k}\right)$ is the coordinate of the *kth* anchor.

It can be seen that $d_{ik}-{\bar{d}}_{ik}$ is the error between the actual distance and the estimated distance. Therefore, the goal of the optimization model (i.e., Equation (1) is to minimize the total error from the unknown node to all anchors.

Recently, SIAs, such as particle swarm optimization algorithm (PSO) [10], cuckoo search algorithm (CS) [11] and bat algorithm (BA) [12], have been successfully applied to achieve higher localization accuracy [13,14,15,16]. However, the existing SIAs optimized solutions cannot achieve a better balance among high localization accuracy, fast convergence and scalability. Among all SIAs, PSO is now prevailing due to its simplicity, its easy yet effective implementation, and it has been widely applied to optimize node localization in WSN [15,16,17,18,19]. However, PSO cannot guarantee a global optimal solution, and hardly adjust to the best results due to its many control parameters. QPSO is an improved PSO variety which outperforms the original PSO in search ability and fewer control parameters [20]. Recently, QPSO has also been used to improve localization accuracy [21,22].

To improve previous works, we proposed a novel range-free localization scheme for static WSNs (called LMQPDV-hop). In LMQPDV-hop, by using a defined weighted factor, a more reasonable distance estimation method for nodes was proposed. Meanwhile, QPSO is reconstructed with the memetic algorithm and Lévy flight to enhance the localization accuracy. In addition, in order to evaluate the localization precision, we compared our LMQPDV-hop to 7 other well-known localization schemes (SIAs based vs. non SIAS based or DV-Hop based vs. non DV-Hop based) in regular and irregular WSN scenarios. The main contributions of this paper are summarized as follows:

(1) A new optimization algorithm (LMQPSO) for node localization in WSN was reconstructed, in which we designed a fast search rule to speed up the convergence of LMQPSO. In addition, to escape from local optimum, MA and Lévy flight were both employed to enhance the global search ability. Furthermore, some other small operations, such as memory mechanism, were adopted to help to find the global solution.
(2) By analyzing the error in the distance estimation of DV-Hop, we modified the calculating method of the average hop distance to reduce the error of the estimated distance among anchors.
(3) By using LMQPSO to optimize the estimated distance, we provided a new efficient localization solution (LMQPDV-hop). LMQPDV-hop was compared to other competitive algorithms according to different densities of anchors and node communication ranges. The results confirm that the new algorithm achieved faster convergence and higher localization accuracy.

The rest of this paper is organized as follows: Section 2 presents the related works on the existing SIA-based localization schemes. The new optimization algorithm (LMQPSO) for node localization is reconstructed in Section 3. In Section 4, we detailed the new localization algorithm based on LMQPSO (LMQPDV-hop). The simulation results and comparisons are illustrated in Section 5. Finally, Section 6 presents the conclusions.

## 2. Related Work

Chowdhury et al. reviewed localization techniques for WSNs [23]. Halder et al. gave a detailed survey of mobile assisted localization algorithms [24]. Here, we summarized the SIAs based schemes.

Generally, node localization consists of two steps: one is distance estimation and the other is coordinate estimation. When using these two steps to determine the coordinate of unknown nodes, error is inevitable, and the smaller the error, the higher the location accuracy. Therefore, in order to improve the location accuracy, the SIA-based localization solution can be studied from the following two aspects: the first is to try to reduce the estimated distance error among anchors and unknown nodes, and the second is to enhance optimization coordinate estimation to find the best result. The second step can be further described as improving the localization optimization model and enhancing the SIAs according to the WSN scenario. In fact, most of the existing research focuses on either one or both of them.

PSO-based localization solutions for WSNs are surveyed in [19], and most of them focused only on the parameter selection and performance comparison of PSO. A weighted PSO DV-Hop (WPDV-hop) was proposed to improve the localization accuracy [16], in which the average hop distance was modified by a weighted factor, and the original PSO was improved by discarding the worst particle far away from the optimal solution in each iteration. However, discarding the worst particle causes PSO to trap into local optimum. In order to further increase the convergence speed and improve the localization accuracy, a new PSO-based scheme (PSOPF) was proposed [17] in which the optimization model described in Equation (1) was adjusted by using a penalty function with an error correction factor. Here, the error correction factor was used to reflect the average error of the estimated distance between the unknown node and the anchor. Though the convergence of PSOPF is faster than that the original PSO, it still suffers from its premature. In the PSO-based localization method for UWSNs (MP-PSO) [15], the range-based PSO, which is used to locate the beacon nodes, still easily falls into local optimum. A PSO-based real-time 3D localization algorithm for indoor UWB WSN was proposed [18], in which the 2D optimization model was extended to 3D model. Meanwhile, the inertia factor and self-cognitive component of the original PSO were also discarded. Thus, this algorithm can easy be trapped in premature. A distributed two-phase PSO algorithm was proposed to solve the flip ambiguity problem of localization in WSNs [25]. This study adopted the general optimization mode. Besides, in order to speed up the convergence, the initial search space of the PSO is reduced by using a bounding box method. However, this work still suffers from its premature convergence. A multi-objective PSO was used to solve the multi-objective optimization localization issues in wireless sensor networks [26]. The multi-objective function consists of the space distance constraint and the geometric topology constraint. This solution offers considerable improvements in localization accuracy and convergence speed, but also has a higher computational complexity and requires additional memory space. QPSO outperforms PSO due to its better global searching ability and fewer control parameters. Thus, QPSO was also adopted to optimize localization for WSNs. For example, original QPSO was used to improve the sequence-based localization for underground coal mine [21]. A new localization algorithm based on improved QPSO (MMQPDV-Hop) was proposed for static WSN [27]. In this scheme, the average hop distance of DV-Hop was modified by a defined distance error weight coefficient, and the QPSO was also improved by discarding the bad particles and utilizing the previous search history of *Mbest* to enhance search ability. However, similarly to WPDV-hop [16], discarding the bad particles can also lead to premature convergence of QPSO.

Besides PSO and QPSO, other SIAs were also applied to solve localization problem in WSNs. CS was used to estimate the sensor’s position [28]. Although the Lévy flight mechanism can help CS to avoid the local optimum with high probability, it is still limited by its many control parameters and slow convergence. A novel oriented CS with Lévy distribution and Cauchy distribution was designed to improve the localization precision of DV-Hop for cyber–physical systems [14], in which the formulation of a new nest created by a host bird was modified to enhance the exploitation capability and the hybrid distribution was employed to improve the global search capability. Another effective CS was designed for node localization in WSN [29]. In this study, the step size of the Lévy flight was modified to approach global optimal solution rapidly, the fitness of each solution was employed to build mutation probability for avoiding local convergence, and the population was restricted in the certain range to prevent the energy consumption caused by insignificant search. An improved BA was used to improve the localization performance of DV-Hop [13]. In this scheme, the average hop distance was modified. Meanwhile, a nonlinear dynamic inertial weight strategy was presented to extend the global search scope and improve local search accuracy, and a new updated solutions strategy was developed to avoid premature convergence.

## 3. Preliminaries

Here, we provided a brief review of PSO, Memetic algorithm (MA), and Lévy flight.

### 3.1. PSO

PSO is a population-based stochastic searching algorithm which is inspired by the social behavior of bird flocking and fish schooling works with a swarm of a predefined size (say *N_P_*) of particles [10]. PSO works by having a population (called a swarm) of candidate solutions (called particles). These particles are moved around in the search-space according to a fitness function. The movements of the particles are guided by their own best-known position in the search-space, as well as the entire swarm’s best-known position. When improved positions are discovered, these then come to guide the movements of the swarm. The process is repeated, and by doing so, it is hoped, but not guaranteed, that a satisfactory solution will eventually be discovered.

In PSO, each particle has a position vector and a velocity vector. The position vector gives a complete candidate solution for the optimization problem, and the velocity vector denotes the position changing tendency. Each particle is evaluated by a fitness function to judge the quality of the solution to the problem. To search the optimal solution, a particle iteratively updates its flying velocity and current position according to its own flying experience, i.e., personal best, called *Pbest*, and according to the flying experiences of the other particles, i.e., global best, called *Gbest*.

In canonical PSO, a particle updates its position and velocity using the following simple rules:

(3) $$V_{i}\left(t+1\right)=w\times V_{i}\left(t\right)+c_{1}\times r_{1}\times \left(Pbest_{i}-X_{i}\left(t\right)\right)+c_{2}\times r_{2}\times \left(Gbest-X_{i}\left(t\right)\right)$$

(4) $$X_{i}\left(t+1\right)=X_{i}\left(t\right)+V_{i}\left(t\right)$$

where $V_{i}=\left\{v_{i}^{1},v_{i}^{2}\ldots ,v_{i}^{D}\right\}$ and $X_{i}=\left\{x_{i}^{1},x_{i}^{2}\ldots ,x_{i}^{D}\right\}$ are the velocity vector and the position vector of the ith particle (say $P_{i}$). *w* is the inertial weight. *t* is the current iteration and *t* + 1 is the next iteration. *c*_1_ and *c*_2_ are acceleration factors termed as cognitive and social components. *r*_1_ and *r*_2_ are two different uniformly distributed random numbers in the range [0,1]. $Pbest_{i}=\left\{Pbest_{i}^{1},Pbest_{i}^{2},\cdots ,\;Pbest_{i}^{D}\right\}$ is the personal best position of $P_{i}$, and $Gbest=\left\{gbest^{1},gbest^{2},\cdots ,gbest^{D}\right\}$ is the global best position of the whole swarm.

### 3.2. Memetic Algorithm

In computer science and operations research, a memetic algorithm (MA) is an extension of the traditional genetic algorithm. It uses a local search technique to reduce the likelihood of the premature convergence. Generally, MAs are a specification of Memetic Computing (MC). MC is the paradigm that uses the notion of memes. In general terms, memes are problem solvers. In MC, memes are included in a global framework, allowing them to cooperate and/or compete in the problem solving. MAs can also be considered as a special class of heuristic search methods inspired by evolutionary theory, which has the idea of “divide to conquer” and the remarkable characteristic is that all memes are allowed to gain some experience through a local search before being involved in the evolutionary process. In other words, MA separates the exploration effort from the exploitation effort in two components.

### 3.3. Lévy Flight

A Lévy flight, named after French mathematician Paul Lévy, is a random walk in which the step-lengths have a probability distribution that is heavy-tailed. Loosely speaking, a random walk is a random process which consists of taking a series of consecutive random steps. Let *S_N_* denote the sum of each consecutive random step *X_i_*, then *S_N_* forms a random walk.

(5) $$S_{N}=\sum_{i=1}^{N}X_{i}=X_{i}+X_{i}+\ldots +X_{N}=\sum_{i=1}^{N-1}X_{i}+X_{N}=S_{N-1}+X_{N}$$

where *X_i_* is a random step drawn from a random distribution, that is, the next state *S_N_* will only depend on the current existing state *S_N-1_* and the motion or transition _XN_ from the existing state to the next state. Here, the step size or length in a random walk can be fixed or varying.

When defined as a walk in a space of a dimension greater than one, the steps made are in isotropic random directions. Later researchers have extended the use of the term “Lévy flight” to include cases where the random walk takes place on a discrete grid rather than on a continuous space. Lévy flights are, by construction, Markov processes. For general distributions of the step-size, satisfying the power-like condition, the distance from the origin of the random walk tends, after a large number of steps, toward a stable distribution due to the generalized central limit theorem, enabling many processes to be modeled using Lévy flights. Many natural phenomena and physical phenomena, such as the flying of albatross, fluorescent particle diffusion, noise, etc., are known to follow Lévy flight (or motion). The random walks of Lévy flight are distributed according to Lévy stable distribution. This distribution is a simple power-law formula: L(s) ~ |s|−1−β where 0 < β < 2 is an index. Mathematically, a simple version of Lévy distribution can be defined as

(6) $$L\left(s,\gamma ,\mu \right)=\left\{ \begin{array}{cc} \sqrt{\frac{\gamma }{2\pi }}\exp\left[-\frac{\gamma }{2\left(s-\mu \right)}\right]\;\frac{1}{{\left(s-\mu \right)}^{3/2}} & \mathrm{if}\;0<\mu <s<\infty \\ 0 & \mathrm{if}\;s\leq 0 \end{array}\right.$$

where the u parameter is the location or shift parameter and the *γ* > 0 parameter is the scale (controls the scale of distribution) parameter.

In general, Lévy distribution should be defined in terms of Fourier transform.

(7) $$F\left(k\right)=\exp\left[-\alpha {\left|k\right|}^{\beta }\right],\;0<\beta \leq 2$$

where α and β are two parameters characterizing this distribution. α∈[−1,1] and controls the scale of distribution. β∈(0,2] and controls the shape of the probability distribution. The analytic form of the integral is not known for general β except for a few special cases. One special case is that when β = 1, the above distribution is equivalent to the Cauchy distribution; another special case is that when β = 2, it corresponds to Gaussian distribution. When β<2, the Lévy distribution is similar to the Gaussian distribution, but with fatter tails, and smaller β causes the distribution to make longer jumps since there will be a longer tail.

For random walks, the Lévy random numbers, i.e., the step length *S*, can be generated as follows:

(8) $$S=\frac{u}{{\left|v\right|}^{1/\beta }}$$

where *u* and *v* are drawn from normal distributions. That is

(9) $$u∼N\left(0,\sigma_{u}^{2}\right),\;v∼N\left(0,\sigma_{v}^{2}\right)$$

where

(10) $$\sigma_{u}={\left\{\frac{\Gamma \left(1+\beta \right)\sin\left(\pi \beta /2\right)}{\Gamma \left[1+\beta /2\right]\beta 2^{\left(\beta -1\right)/2}}\right\}}^{1/\beta }$$

Then the step size can be calculated as

(11) stepsize=0.01×S

## 4. LMQPSO: The Proposed QPSO with the Memetic Algorithm and Lévy Flight

Since our localization optimization model can be formalized as a binary quadratic function (see Section 5.2.2 for detailed information) and QPSO has great advantages in solving such optimization problems, therefore, QPSO was employed to optimize the coordinates of unknown nodes in this paper. In the WSN scenario, the coordinates of unknown nodes should be estimated as quickly as possible, thus the quick convergence speed and a good global solution search ability of QPSO are both required. However, QPSO still has a slower convergence speed and easily falls into local optimum, especially for large-scale optimization problems. Thus, the original QPSO should be improved. To overcome these drawbacks of QPSO, an improved QPSO with MA and Lévy flight (LMQPSO) was proposed to optimize the coordinate of unknown nodes in WSN.

Since QPSO is inspired by quantum mechanics, it changes the search strategy of the PSO [10] and discards the velocity update step. Its search strategy and particle status update rule are as follows:

(12) $$X_{i}^{j}(t+1)=C_{i}^{j}\left(t\right)\pm \beta *|Mbest^{j}\left(t\right)-X_{i}^{j}\left(t\right)|*\ln\left(\frac{1}{u_{i}^{j}(t+1)}\right),\;u_{i}^{j}\left(t+1\right)=rand\left(0,1\right)$$

where $X_{i}=\left\{x_{i}^{1},x_{i}^{2},\cdots ,x_{i}^{D}\right\}$ is the ith particle’s position (say *P_i_*), and *D* is the dimension of the target search space. $C_{i}^{j}\left(t\right)$ is the local attractor point of *P_i_*, which is defined as

(13) $$C_{i}^{j}\left(t\right)=\alpha *Pbest_{i}^{j}\left(t\right)+\left(1-\alpha \right)*Gbest^{j}\left(t\right),\;\alpha ∼U\left(0,1\right)$$

where $Pbest_{i}^{j}$ and $Gbest^{j}$ are the *jth* dimensional of $Pbest_{i}$ and Gbest.

*Mbest* in Equation (12) is a global point which is used to calculate the next generation iterator variant. It is defined as the average of the personal best positions of all particles:

(14) $$\begin{array}{cc} Mbest\left(t\right) & =\left(Mbest^{1}\left(t\right),Mbest^{2}\left(t\right),\ldots ,Mbest^{D}\left(t\right)\right)=\frac{1}{N_{p}}\sum_{i=1}^{N_{p}}Pbest_{i}\left(t\right)\\ & =\left(\frac{1}{N_{p}}\sum_{i=1}^{N_{p}}Pbest_{i}^{1}\left(t\right),\frac{1}{N_{p}}\sum_{i=1}^{N_{p}}Pbest_{i}^{2}\left(t\right),\cdots ,\frac{1}{N_{p}}\sum_{i=1}^{N_{p}}Pbest_{i}^{D}\left(t\right)\right) \end{array}$$

where $N_{p}$ is the population size.

β in Equation (12) is the contraction–expansion coefficient. This control parameter can be tuned to control the convergence speed of the algorithm.

To speed up the convergence and enhance the global searching ability, this paper reconstructed the original QPSO by introducing MA and Lévy flight mechanisms. MA was employed as the skeleton to enhance the global search ability and Lévy flight was adopted to help particles escape from local optimum.

The new skeleton of LMQPSO consists of three components: a local search rule for finding the optimum in each subgroup, a global search rule for searching for the optimal solution over all the subgroups, and a shuffling process for enhancing the diversity. For the local search rule, a fast search rule was newly designed to accelerate the convergence. Moreover, the previous search history of *Mbest* and Lévy flight was used to help particles to escape from the local optimum. The new LMQPSO is detailed in the following section.

### 4.1. Skeleton of LMQPSO

The skeleton of LMPSO is shown from line 3 to line 30 in Algorithm 1. Based on the ideal of MA, the particle swarm was divided into m subgroups, and a local search strategy was used in each subgroup (line 7~line 28). The global search strategy described in line 29 was used to integrate all the local information of each subgroup to find the global optimal solution. In LMQPSO, the sorting and grouping operations in line 4 are very important. The former is used to switch local search information and the latter affects the global search result. To balance the search space of each subgroup, our grouping operation is detailed as follows: firstly, the population was sorted in descending order by the fitness values of the particles. Obviously, the last particle in the sorted population had the smallest fitness value, which means that it was the *Gbest* in the population. Then, the sorted population was divided into *m* subgroups, each subgroup containing n particles, where n = $N_{p}/m$. The *ith* particle was assigned to the *jth* subgroup, where j = i mod *m*.
**Algorithm 1.** pseudo code of LMQPSOInput: *N_p_*: number of particles;   *D*: the dimension of optimal problem;   *Max_It*: the maximum iteration   *X_max*,*X_min*: the rang of particle positionOutput: *Gbest*1. Randomly initialize all particles, $X_{i}=\left\{x_{i}^{1},x_{i}^{2}\ldots ,x_{i}^{D}\right\}$, for i = 1, 2, …, *N_p_*:2. Evaluate the fitness values; set *X* to be Pbest3. *while iter < Max_It*4. Sort all particles (descending) and divide them into *m* subgroups // seeing 4.1 for detail.5.  *Gbest* = *X_N_p__*;6.  *Mbest’*(*t*) = 0;7. *for k = 1 to le   // Number of previous history memorized by Mbest*’. the local search begin8.  *for i* = 1 to *m*    // *For each subgroup*9.   Search the local *Gbest’*(*t*);10.   Calculate the local attractor *C’*(*t*);    //Using Equation (15)11. Calculate the local *Mbest’*(*t*);      //Using Equations (18)~(19)12. *for j* = 1 to *N_p_*          //*Update each particle’s position in this subgroup*13.  *if rand*>=0.514.   Calculate $X_{j}^{\prime }\left(t+1\right)$ Using Equation (20)15.  *else*16.   Calculate $X_{j}^{\prime }\left(t+1\right)$ with Lévy flight //Using Equation (21)17.  *end if*18.  Bound $X_{j}^{\prime }\left(t+1\right)$ within [*X_max_*,*X_min_*]19.  *If*
$X_{j}^{\prime }\left(t+1\right)$ is better than $X_{j}\left(t\right)$   //*Discarding the worse particle*20.   Update $X_{j}\left(t+1\right)=X_{j}^{\prime }\left(t+1\right)$21.  *else*22.   Update $X_{j}\left(t+1\right)=X_{j}\left(t\right)$23.  *end if*24.   *end for*  // *j*25.  *end for*  //*i*26. *Pbest* = *X*27.   *Iter* = *iter+1*28. *end for*    //*k Finished the local search*29. *Gbest* = *X_i_ if X_i_* has the best fitness value//Global search to find the best solution30. *end while*


### 4.2. Fast Local Search Rule

To speed up the convergence, we redefined the core components of the position update rule in the original QPSO (i.e., Equation (12)) to design a fast local search rule which was used in the local search step of LMQPSO.

#### 4.2.1. New Local Attractor C_i_ for Subgroup

In LMQPSO, the searching rule of the original QPSO was used to find the *Gbest* in each subgroup (called local *Gbest*), and the original local attractor *C_i_* was used to guide particles to a better position by taking advantage of the integrated knowledge of *Pbest*’ and *Gbest*’ in the subgroup. To accelerate this guide process, we discarded *Pbest*’ and kept the local *Gbest*’ of the subgroup to attract particles to a better position. That is,

(15) $$C_{i}^{\prime }=Gbest^{\prime }$$

Obviously, guiding particles only by *Gbest*’ may fall into the local optimum, but MA and the Lévy flight can help particles to jump out the local optimum.

#### 4.2.2. New Definition of *Mbest* for Subgroup

Equation (14) shows that *Mbest* represents the swarm knowledge and is also used to guide particles flying in a better position. However, it is well known that the swarm intelligence not only contains the *Pbest* knowledge of individual particles, but also contains the *Gbest* of the whole swarm. Therefore, the definition of *Mbest* in Equation (14) is incomplete. Luckily, the original local attractor $C_{i}^{j}\left(t\right)$ (i.e., Equation (13)) in QPSO contains both *Pbest* and *Gbest* of the swarm, thus it can accurately represent the knowledge of swarm intelligence. Therefore, we redefined the *Mbest* for each subgroup as follows:

(16) $$\begin{array}{cc} Mbest^{\prime }\left(t\right) & =\left(Mbest^{1}\left(t\right),Mbest^{2}\left(t\right),\ldots ,Mbest^{D}\left(t\right)\right)=\frac{1}{N_{p}}\sum_{i=1}^{N_{p}}C_{i}\left(t\right)\\ & =\left(\frac{1}{N_{p}}\sum_{i=1}^{N_{p}}C_{i}^{1}\left(t\right),\frac{1}{N_{p}}\sum_{i=1}^{N_{p}}C_{i}^{2}\left(t\right),\cdots ,\frac{1}{N_{p}}\sum_{i=1}^{N_{p}}C_{i}^{D}\left(t\right)\right) \end{array}$$

#### 4.2.3. Discarding Worse Particles

In each iteration of QPSO, the new *X*(*t* + 1) can be divided into two parts:

(17) $$X\left(t+1\right)=\left\{ \begin{array}{cc} X_{Better}\left(t+1\right) & if\;fit\left(X\left(t+1\right)\right)<fit\left(X\left(t\right)\right)\\ X_{Worst}\left(t+1\right) & otherwise \end{array}\right.$$

where fit(X(t)) is the fitness value of X(t), that is, $X_{Better}\left(t+1\right)$ is better than X(t) and $X_{Worst}\left(t+1\right)$ is not better than *X*(*t*).

Clearly, $X_{Worst}\left(t+1\right)$ can not generate a better solution than $X_{Better}\left(t+1\right)$. Therefore, we discarded them and kept the better particles only in the next iteration.

#### 4.2.4. Synchronous Search for Each Dimension

QPSO randomly searches for each dimension of the full space, which results in its poor performance, especially in a high-dimensional space. Moreover, the search time increases sharply as the number of dimensions increases [20]. Therefore, a synchronous search method was adopted to accelerate the convergence speed, that is, using the same random search step in each dimension to enhance the exploring ability. Namely, $u_{i}^{j}\left(t+1\right)$ in Equation (12) was replaced by u(t+1).

### 4.3. Local Search with the Previous History Memorized by our New $Mbest^{\prime }$

In QPSO, only the current *Pbests* and *Gbest* were integrated in $Mbest^{\prime }$ for each of its search, whereas the previous *Pbests* and Gbest were discarded. The new fast search rule can quickly find the better position, but at the same time, it may also fall into the local optimal solution. Authors in [20,30] reported that previous search history can enhance the global search performance. Therefore, in this paper, we let $Mbest^{\prime }$ not only integrate the current *Pbests* and *Gbest*, but also the previous *Pbests* and *Gbest*. Thus, the $Mbest^{\prime }$ was further improved as follows:

(18) $$Mbest_{i,j}^{\prime }\left(t\right)=\frac{1}{N_{p}}*\left(Mbest_{i,j}^{\prime }\left(t-1\right),\sum_{i=1}^{N_{p}}C_{i,1}\left(t\right),\sum_{i=1}^{N_{p}}C_{i,2}\left(t\right),\ldots ,\sum_{i=1}^{N_{p}}C_{i,D}\left(t\right)\right)$$

(19) $$Mbest_{i,j}^{\prime }\left(0\right)=\frac{1}{N_{p}}*\left(\sum_{i=1}^{N_{p}}C_{i,1}\left(0\right),\sum_{i=1}^{N_{p}}C_{i,2}\left(0\right),\ldots ,\sum_{i=1}^{N_{p}}C_{i,D}\left(0\right)\right)$$

Taken together, the above four improvements, Equation (12) was reconstructed as

(20) $$X_{i}^{j}(t+1)=C_{i}^{\prime }{}^{j}\left(t\right)\pm \beta *|Mbest^{\prime }{}^{j}\left(t\right)-X_{Better}{}_{i}^{j}\left(t\right)|*\ln\left(\frac{1}{u(t+1)}\right),\;u\left(t+1\right)=rand\left(0,1\right)$$

It is worth noting that when the number of the subgroup and the number of the previous history memorized by $Mbest^{\prime }$ are both set to 1, LMQPSO is like the standard QPSO.

### 4.4. Position Update with Lévy Flights

To accelerate the convergence, we redefined the four core components of the original QPSO. However, all these methods may lead our new algorithm to be trapped into a local optimal solution. Although searching with memory(*Mbest*’) can direct particles to better position, a more powerful mechanism is still required to help particles jump out of the local optimum. Therefore, we introduced the Lévy flight method into our algorithm to achieve this goal.

In our new mechanism, each particle updated its position according to a random probability. If the random value was greater than or equal to 0.5, the position of the particle was updated, as seen in Equation (20), otherwise, the position of the particle was updated as follows:

(21) $$X_{i}^{j}(t+1)=Levy_{flight}\left(C_{i}^{\prime }{}^{j}\left(t\right)\right)\pm \beta *|Mbest^{\prime }{}^{j}\left(t\right)-Pbest_{i}^{j}\left(t\right)|.\ln\left(\frac{1}{u(t+1)}\right),\;u\left(t+1\right)=rand\left(0,1\right)$$

where

(22) $$Levy_{fight}\left(C_{i}^{\prime }\left(t\right)\right)=Gbest^{\prime }+rand(1,D)⊗\mathrm{step}$$

where *Gbest*’ is the best solution of the subgroup, and the step can be calculated as follows:

(23) $$\mathrm{step}=0.01\times \mathrm{stepsize}⊗Gbest^{\prime }(t)$$

Here, the step size can be calculated using Equation (23). ⊗ is the element-by-element multiplication.

## 5. LMQPDV-Hop: The Proposed Localization Algorithm Based on LMQPSO and DV-Hop

**Figure 1:**
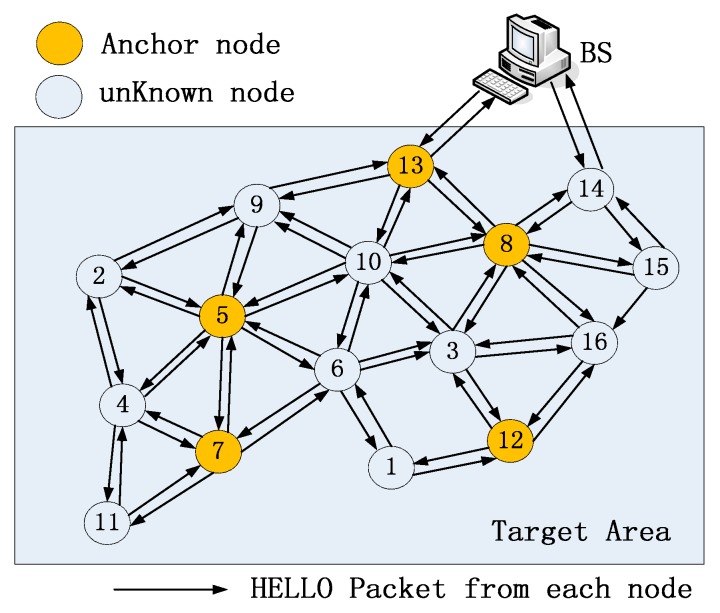
Illustration of gathering the information of nodes by BS.

In this paper, a new centralized localization algorithm, namely LMQPDV-hop, was proposed to solve the static node localization problem in WSNs. In LMQPDV-hop, DV-Hop was employed as an underground mechanism to estimate the distances between unknown nodes and anchors. Next, these estimated distances were optimized and converted to the coordinates of the unknown nodes by LMQPSO. Considering the resource constrains (e.g., small storage, low computation and energy) of sensor nodes, the new algorithm is run by a base station (BS). In the paper, we assumed such a WSN scenario: N unknown nodes and M anchors were randomly deployed in the regular or the irregular target sense area. Each node had a transmission range (R). The individual node only owned the local information, such as its unique ID, coordinate (only anchor has its initial value), and the hops to each anchor. Each node sent its data through multi-hops to BS. BS has enough resource (e.g., storage, computation, energy) to complete the new localization algorithm. This procedure of BS gathered the information of all the nodes, as Figure 1 illustrates.

### 5.1. Gathering Estimation Distances

#### 5.1.1. Estimating Distance by DV-Hop

(1) At the beginning, all nodes undergo bootstrapping process, in which each anchor broadcasts a HELLO packet $\left\{{\mathrm{ID}}_{k},<x_{k},y_{k}>,hop\_count\right\}$ that contains its node id, coordinate (initiate value is not null for anchors) and hop count (initiate value is 0) to the network. When a node receives the packet, it records the ID, the coordinate and the smallest hop count to each anchor, then it forwards the packet to its neighbors after hop values plus 1 until the packet reaches BS. In this way, all the nodes gather the smallest hop count to anchors.
(2) When the BS receives all the packets from the network, it estimates the distances from each unknown node to all the anchors by using the following equation:
  (24) $$dis_{ij}=h_{ij}\times HopSize_{i}$$
  where $dis_{ij}$ is the estimated distance between the jth unknown node and the ith anchor, $h_{ij}$ is the smallest hop count, and $HopSize_{i}$ is the average hop distance of the anchor and can be given by
  (25) $$HopSize_{i}=\frac{\sum_{i\neq j}\sqrt{{\left(x_{i}-x_{j}\right)}^{2}+{\left(y_{i}-y_{j}\right)}^{2}}}{\sum_{i\neq j}h_{ij}^{\prime }}$$
  where $h_{ij}^{\prime }$ is the smallest hop between anchor i and anchor j(i≠j) and $\left(x_{i},y_{i}\right)$ is the coordinate of the anchor.

#### 5.1.2. Improvement of the Estimated Distance

However, due to the error of the estimated distance, the localization accuracy of the original DV-Hop is always unsatisfactory. The essential reason is the inaccuracy of the average hop distance (*HopSize_i_*) in Equation (13). For example, there is a possibility that the real distance between two anchors is long but the Euclidean distance is short (e.g., in a built-up or hilly area), thus the $HopSize_{i}$ calculated by using Equation (25) is smaller than the real distance, which results in a smaller estimated distance. Therefore, it is crucial to design improvements to acquire a more accurate *HopSize_i_*. Additionally, we can observe that the distance error accumulates as the number of hop among nodes increases. That is, the estimated distance between nodes with small hop count is more accurate than that between nodes with large hop count. Therefore, the impact of the anchor with small hop count to the unknown node should be reduced to help to improve the accuracy of the estimated distance. Therefore, the hop count must be considered in the improved calculation method of *HopSize_i_*.

The ideal of modifying the $HopSize_{i}$ is as follows: the distance error between anchors and the hop count from the unknown nodes to the anchors were both considered to adjust the $HopSize_{i}$. That is, a normalized weighing factor was introduced to modify the average hop distance. The weighing factor can be normalized as

(26) $$\lambda_{ij}=\frac{\frac{1}{H_{i}\times e_{ij}^{2}}}{\sum_{k=1}^{M}H_{k}\times e_{ik}^{2}}$$

where $e_{ij}$ is the distance error between anchors *i* and *j*, which is defended as

(27) $$e_{ij}=d_{est}^{i,j}-d_{true}^{i,j}=d_{est}^{i,j}-\sqrt{{\left(x_{i}-x_{j}\right)}^{2}+{\left(y_{i}-y_{j}\right)}^{2}}$$

where $d_{est}^{i,j}$ is the estimated distance which is calculated by Equation (24). $d_{true}^{i,j}$ is the actual Euclidean distance. *H_i_* is the hop count from the unknown node to *ith* anchor. *H_k_* is the hop count from the unknown node to *kth* anchor. We can see from Equation (27) that the larger *H_i_* and $e_{ij}$ are, the smaller $\lambda_{ij}$ is. It is well known that the estimated distance between anchors with larger hop count produces bigger errors. Hence, the large hop count requires a smaller weighting factor, while the small hop requires a larger weighting factor. Accordingly, the *HopSize_i_* in Equation (25) can be improved as

(28) $$Min\left(f(x,y)=\sum_{i=1}^{M}{\left(\sqrt{{\left(x-x_{i}\right)}^{2}+(y-y_{i}}\right)}^{2}-d_{i}{)}^{2})\right)$$

Equation (28) shows that the anchors near the unknown node and the anchors with a smaller distance error have larger weight and greatly affect the *HopSize_i_*, whereas the anchors far away from the unknown node and the anchors with bigger distance error have a smaller effect on *HopSize_i_*. Therefore, the error caused by the estimated distance was reduced using Equation (28).

### 5.2. Optimization Localization Result

After estimating the distances among all the nodes, BS then runs the proposed LMQPSO to determine the coordinates of the unknown nodes. The detailed process includes particle initialization and fitness function determination, followed by the position update phase, as shown below.

#### 5.2.1. Initialization of Particles

We represented the particles in such a way that each particle provides the coordinate (x, y) of the unknown node with the dimension of 2. We initialized each component *X*_*i*,1_ and *X*_*i*,2_ (1 ≤ *i* ≤ *N_p_*) using the coordinate generated randomly within the target sensor area. For example, the sensor field was rectangular area L*W, then 0 < = *X*_*i*,1_ < = L, 0< = *X*_*i*,2_ < = W.

#### 5.2.2. The Fitness Function Derivation

The fitness function is very important for the optimization algorithm, because it directly affects the final results. We constructed a new fitness function to evaluate the individual particle of the population, which helped us to periodically update the *Pbest* and *Gbest* of swarm. Our objective in the new algorithm was to minimize the total error between the actual distance and the estimated distance from the unknown nodes to anchors. Therefore, the fitness function can be defined as follows:

(29) $$Min\left(f(x,y)=\sum_{i=1}^{M}{\left(\sqrt{{\left(x-x_{i}\right)}^{2}+(y-y_{i}}\right)}^{2}-d_{i}{)}^{2})\right)$$

where (*x*,*y*) is the unknown node’s coordinate,(*x_i_*,*y_i_*) is the *ith* anchor’s coordinate, and *M* is the number of anchor nodes. $\sqrt{{\left(x-x_{i}\right)}^{2}+(y-y_{i}}{)}^{2}$ is the actual distance. *d_i_* is the estimated distance between the unknown node and the *ith* anchor, which is calculated by Equation (24). Obviously, a smaller *f*(*x*,*y*) means a more accurate (*x*,*y*).

Similarly with the distance error among anchors, the bigger the hop count among the nodes the larger the estimated distance error. The distance error of the unknown node should also be weighted by the hop count, and the normalized weight factor is defined as follows:

(30) ωi=1Hi∑k=1M1Hk

where *H_i_* and *H_k_* are the same in Equation (26). Therefore, the fitness function is now improved as

(31) $$f(x,y)=\sum_{i=1}^{M}\omega_{i}{\left(\sqrt{{\left(x-x_{i}\right)}^{2}+(y-y_{i}}\right)}^{2}-d_{i}{)}^{2})$$

#### 5.2.3. Position Update

In the new algorithm, the particles update their positions in each iteration using Equation (20) and Equation (21). It is noteworthy that the algebraic steps of the addition and subtraction operation may cause the new position of the particle to be outside of the target sensor area. Therefore, our algorithm should ensure that the new position can satisfy the range.

If the new position is negative, then replace the position with a newly generated random number that tends to zero.

If the new position is greater than the maximum, then replace the position with the maximum value.

After obtaining the new position, the particle *P_i_* then updates its $Pbest_{i}^{t+1}$ as follows s:

(32) $$Pbest_{i}^{t+1}=\left\{ \begin{array}{ll} X^{t+1}, & \mathrm{if}\;F(X^{t+1})<F(Pbest_{i}^{t})\\ P_{i}^{t}, & otherwise \end{array}\right.$$

and the global best particle $Gbest_{i}^{t+1}$ is updated as follows:

(33) $$Gbest_{i}^{t+1}=\left\{ \begin{array}{ll} Pbest_{i}^{t+1}, & \mathrm{if}\;F(Pbest_{i}^{t+1})<F(Gbest_{i}^{t})\\ Gbest_{i}^{t}, & otherwise \end{array}\right.$$

The positions are iteratively updated until the termination condition is satisfied. In our approach, there are two conditions: a predefined number of iterations and an accuracy requirement. After satisfying one of the two conditions, the particle $Gbest_{i}^{t+1}$ represents the position result.

### 5.3. The Localization Flow of LMQPDV-hop

The objective of our node localization scheme is to find the coordinate of unknown nodes by using *M* anchors. The process followed is described below:

Step 1. All nodes are deployed randomly in the regular or irregular target sensor field. At the beginning of the network initiation, namely, the neighbor discovery phase. The anchors calculate their location awareness and transmit their coordinates to the network. All nodes (anchors and unknown nodes) record the smallest hop counts to anchors and re-broadcast the information of these locations until it reaches the BS.
Step 2. After BS receives all the location packages, it calculates the average distance using Equation 28. Subsequently, all the distances from the unknown nodes to the anchors are estimated using Equation (24).
Step 3. Based on the distance matrix derived from the estimated dances, BS runs the proposed LMQPSO to solve the optimization model (i.e., Equation (31)) and determines the coordinates of unknown nodes.

**Figure 2:**
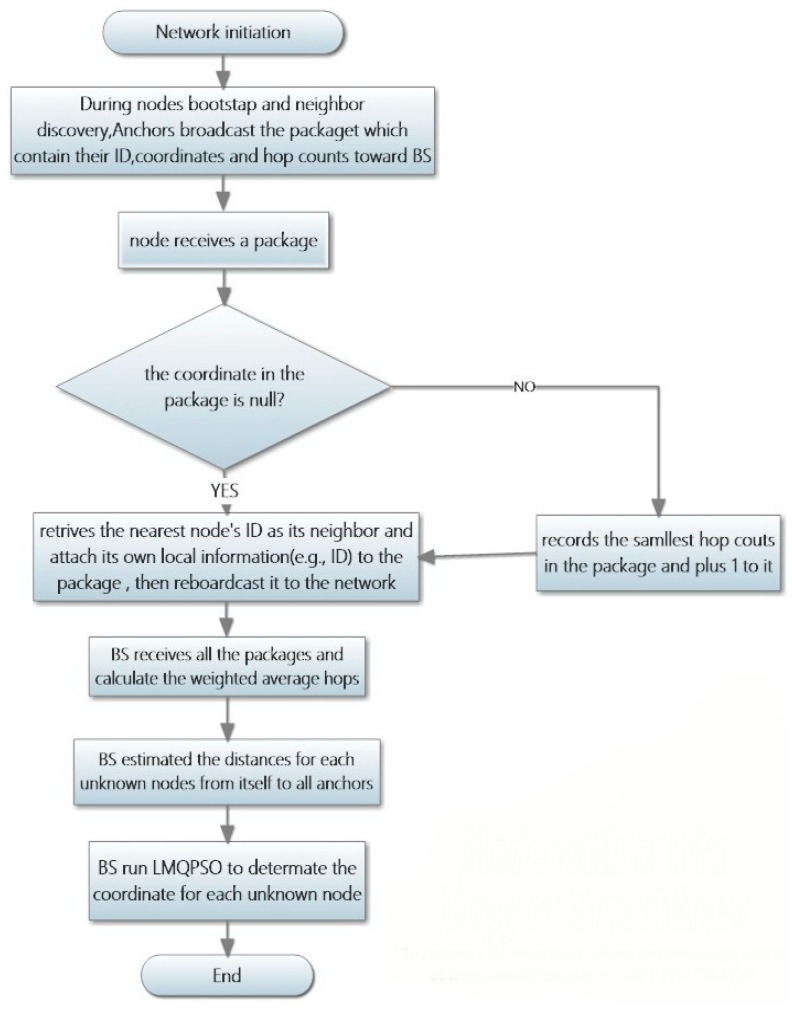
Flowchart of LMQPDV-hop.

The detailed steps of our algorithm are depicted in the flowchart shown in Figure 2.

## 6. Results and Analysis

To validate the LMQPDV-hop, we compared it to some classic rang-free localization algorithms (i.e., such as DV-hop, Centroid, and Grid_Scan) along with the localization algorithms based on SIAs (i.e., WPDV-hop [16], PSOPF [17], CuckooDV-hop [28] and MMQPDV-hop [27]) in terms of location errors and convergence speeds. To comprehensively evaluate the new localization algorithm, we arranged two groups of experiments and two groups of simulations: Experiment 1 was conducted to compare the proposed LMQPSO to other SIAs used by the other SIA based algorithms on four internationally recognized standard benchmark functions (dimensions are 30). For comparison, four other SIAs were used, namely, PSO1 used by WPDV-Hop, PSO2 used by PFPSO, CS used by CuckooDV-hop, and MMQPSO used by MMQPDV-hop. Experiment 2 was performed to evaluate the convergence speed of the above 5 SIAs when they are applied to WSNs. To make a fair comparison in this experiment, we used the same fitness function (i.e., Equation (31)) on the same WSN topology (WSN#1 with 60 anchors). Simulation 1 WAS carried out to evaluate the effect of the number of anchor and the communication range on location error. In Simulation 1, LMQPDV-hop was compared to seven other well-known localization methods, namely DV-hop, Centroid, Grid_scan, WPDV-hop, PSOPF, CuckooDV-hop and MMQPDV-hop, in both the regular sensor area (WSN#1) and the irregular sensor area (WSN#2). In Simulation 2, we compared the localization results of all these eight localization methods used in Simulation 1. In this simulation, the anchor proportion was 30% and the communication range was set to 250.

It is worth noting that in Simulation 1 and Simulation 2, different numbers of anchors in WSN#1 and WSN#2 signifies different WSN topologies, in which the new number of anchors and unknown nodes need to be randomly re-deployed. This is because the total number of all nodes is fixed in the same WSN, and thus the new anchor number also indicates a new unknown node number, which, in turn, means a new network deployment. For this reason, each different anchor number on the x axis in Figures 7–18 actually represents a different WSN topology.

In order to reduce statistical errors in these experiments and simulations, each algorithm was tested independently multiple times and the mean value (Mean), the standard deviations (SD) in all the runs were calculated as the statistics for the performance measures. The Mean represents the global convergence of the algorithm, and the SD represents the stability of the algorithms.

**Figure 3:**
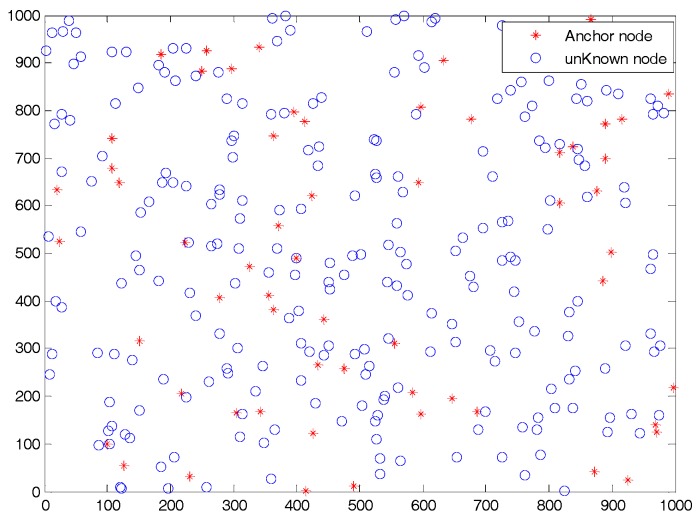
Illustration of deployment for WSN#1.

**Figure 4:**
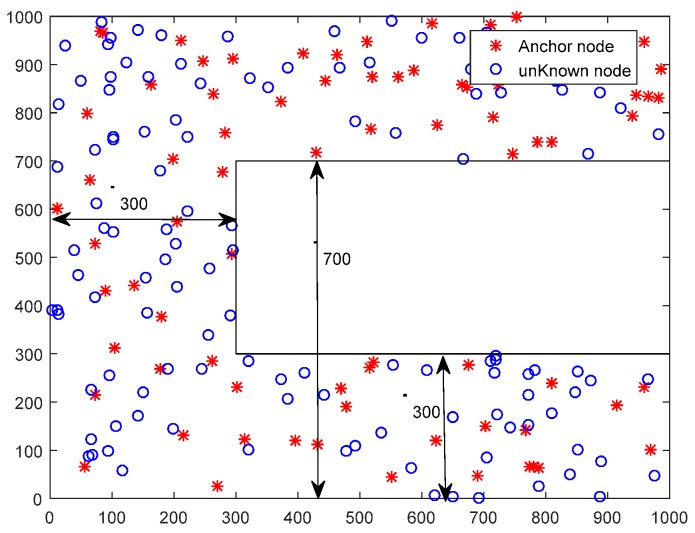
Illustration of deployment for WSN#2.

Simulations were performed on two different WSN deployment scenarios. One was WSN#1, which is illustrated in Figure 3 and all the nodes (i.e., $N_{all}=300$) were randomly deployed in the $1000*1000\;m^{2}$ target sense area. The other was WSN#2, and all the nodes were randomly deployed in the C type area, as shown in Figure 4. The detailed simulation parameters are listed in Table 1.

The same PSO parameters for WPDV-hop as those in literature [16] were set, that is, $c_{1}=c_{2}=2$, $w_{max}=0.95,w_{min}=0.4$, and the weight factor $w=w_{\max}-iter\times \frac{w_{\max}-w_{\min}}{Max\;Iteration}$. In PSOPF, penalty factory M = 8. The *β* value for Lévy flight in LMQPDV-hop and CuckooDV-hop was set to 1.5.

All algorithms were executed 10 times, and the average was taken as the final result. The normalized average location error is defined as follows:

(34) $$NE_{L}=\frac{\sum_{i=M+1}^{N_{L}}\sqrt{{\left({\hat{x}}_{i}-x_{i}\right)}^{2}+{\left({\hat{y}}_{i}-y_{i}\right)}^{2}}}{N_{L}\times R}$$

where *N_L_* is the number of located nodes, (*x_i_,y_i_*) is the calculated coordination of the ith node and $\left({\hat{x}}_{i},{\hat{y}}_{i}\right)$ is its true coordinate. It can be deduced that the number of nodes that can not be located (i.e., $N_{NL}$) can be calculated as follows: *N_NL_* = *N* − *N_L_*.

All the experiments and simulations were done in Matlab platform on Win 7 with Intel core i3-2100 Dual-Core CPU (3.10 GHz) and 4 GB RAM.

### 6.1. Experiment 1

We conducted several experiments to evaluate the performance of LMQPSO and compare it to other optimal algorithms.

Firstly, four well-known test functions were used to evaluate LMQPSO, which have been widely adopted in benchmarking optimization algorithms, namely Sphere, Rastrigin, Rosenbrock, and Griewank, and their detailed information is as follows:

(1) Sphere $f_{1}\left(x\right)=\sum_{i=1}^{d}x_{i}^{2},x_{i}\in \left[-100,100\right],\;Global\;optimum\;is\;0$
(2) Rastrigin $f_{2}\left(x\right)=10d+\sum_{i=1}^{d}\left[x_{i}^{2}-10\cos\left(2\pi x_{i}\right)\right],x_{i}\in \left[-100,100\right],\;Global\;optimum\;is\;0$
(3) Rosenbrock $f_{3}\left(x\right)=\sum_{i=1}^{d-1}\left[\left(1-x_{i}^{2}\right)+100*{\left(x_{i+1}-x_{i}^{2}\right)}^{2}\right],x_{i}\in \left[-100,100\right],\;Global\;optimum\;is\;0$
(4) Griewank $f_{4}\left(x\right)=\frac{1}{4000}\sum_{i=1}^{d}x_{i}^{2}-\prod_{i=1}^{d}cos\left(\frac{x_{i}^{2}}{\sqrt{i}}\right)+1,x_{i}\in \left[-100,100\right],\;Global\;optimum\;is\;0$

Among these functions, both Sphere and Rosenbrock are unimodal functions, which are used to evaluate the solution quality and convergence speed of the optimization algorithm. Rastrigin and Griewank are both multimodal functions which are used to test the global searching ability of optimization algorithm.

**Figure 5:**
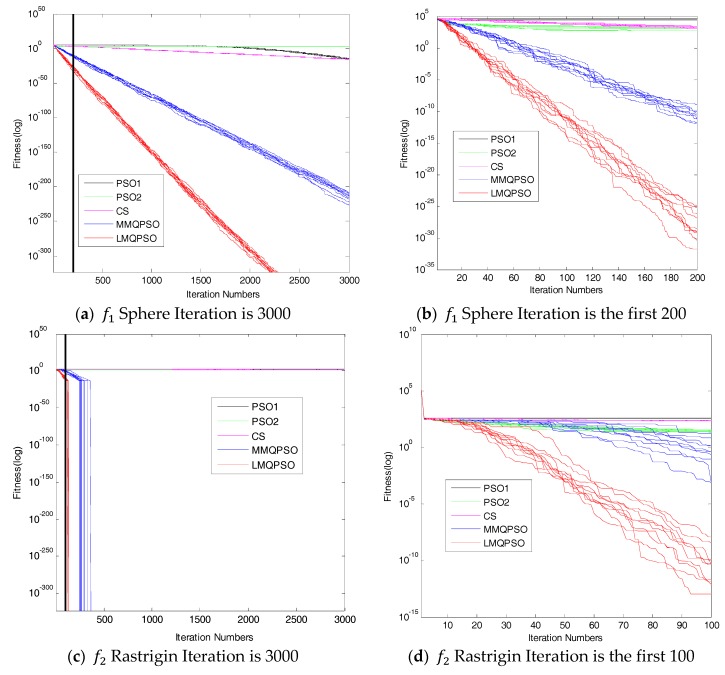

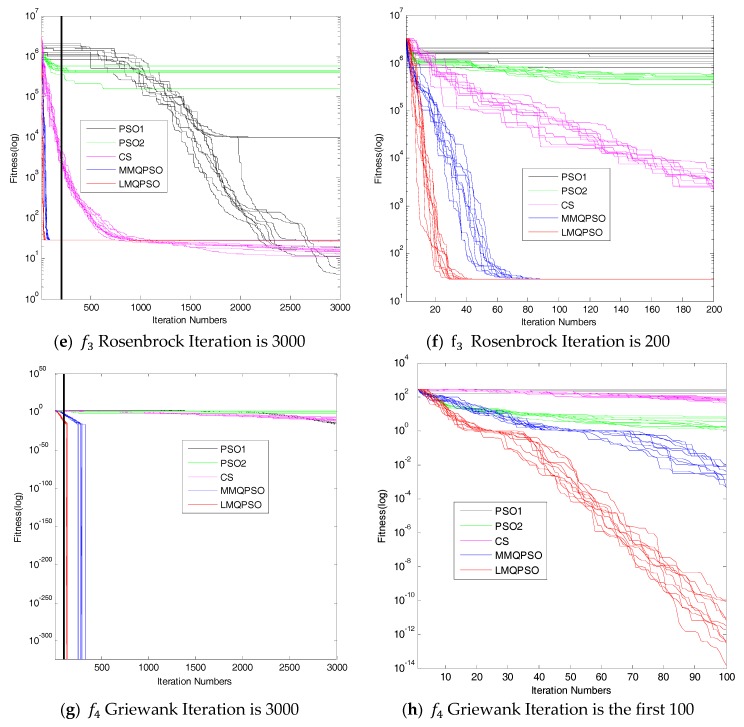
Comparison Results of PSO1, PSO2, CS, MMQPSO and LMQPSO.

The parameters used for this test were: Function dimension D = 30, number of iterations *PGen* = 3000. The simulation results in Figure 5a–c illustrate the evolution of the optimal fitness of these algorithms.

For *f*_1_ (Sphere), Figure 5a,b show that the search improvement of the LMQPSO was the best one. LMQPSO performed best and consistently provided the global result, whereas nothing else could achieve that. The LMQPSO demonstrated the best performance.

For *f*_2_ (Rastrigin), as illustrated in Figure 5c,d, although both LMQPSO and MMQPSO can find the global solution, LMQPSO has a faster convergence speed. In other words, LMQPSO still had the best performance.

The best performance on *f*_1_ and *f*_2_ demonstrates that LMQPSO had good exploration and exploitation capabilities, yet fast convergence. The main contributions were our wonderful algorithm’s framework, the particle update rule and the fast search rule.

*f*_3_ (Rosenbrock) is unimodal in a search space, but it can be treated as a multimodal function in high-dimensional cases. It is difficult for *f*_3_ to achieve global optimum. As Figure 5e shows, none of these optimization algorithms can find the global solution. As shown in Table 2, although CS achieved the best result among these competitive algorithms, the difference between CS and LMQPSO is not obvious. However, as can be seen from Figure 5f, LMQPSO had the fastest convergence speed and the converge speed was much faster than CS. The rapid convergence of LMQPSO is mainly due to our new fast search rule.

*f*_4_ (Griewank) is a rotated multimodal function. It can be used to test the capability of exploring global optimal solution of proposed algorithms. As shown in Figure 5g and Table 2, both MMQPSO and LMQPSO can achieve the global optimum. Figure 5h shows that LMQPSO achieved better value than MMPSO in each iteration. In addition, Figure 5h also illustrates that LMQPSO slightly led to local minimum solution (iteration between 20 and 35), but the Lévy flight and MA mechanism can help it jump out the local optimum quickly. Figure 5g,h demonstrate that LMQPSO is the fastest to find the global optimum, which means that LMQPSO can overcome the shortcomings of converging to the local optimum and improve the global search ability.

Table 2 shows the global mean values and the standard deviation of the five solutions during 10 rounds experiments. It can be observed that LMQPSO almost achieved the best solution on all functions excluding $f_{3}$. These comparisons confirm that the improvements we made to the original QPSO, which include the introduction of MA and Lévy flight mechanism, designing the fast search rule, indeed made LMQPSO perform better than other SIAs in most of the test functions. The reason is that our improvements offered LMQPSO the ability of avoiding local optima and sped-up the convergence. More specifically, this is due to our improvements’ contribution to the capability improvement of diversity and jumping out of likely local optima.

### 6.2. Experiment 2

**Figure 6:**
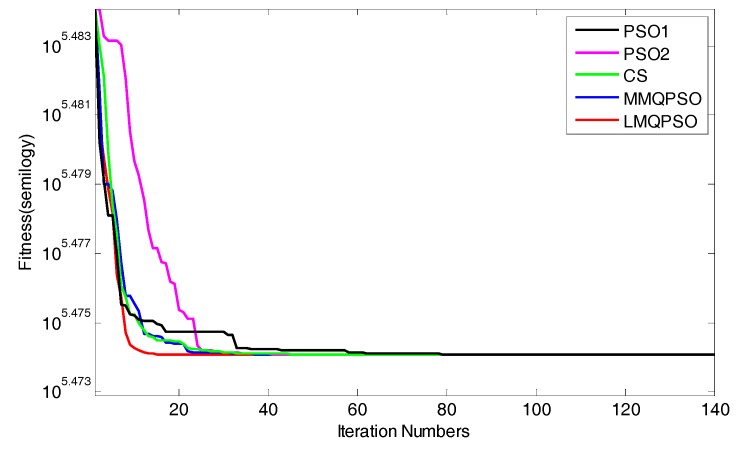
The Convergence of LMQPSO.

Secondly, we evaluated the convergence of the above 5 SIAs when they were applied to WSNs. Figure 6 shows the convergence results. To make a fair comparison, the same fitness function (i.e., Equation (31)) and the same WSN topology (WSN#1 with 60 anchors) were adopted. As shown in Figure 6, as the iterations increased, the fitness values of all algorithms were on the decline and finally stabilized. In order to achieve stability, PSO1 took about 168 iterations, PSO2 took about 47 iterations, CS took about 46 iterations, MMQPSO took about 36 iterations, but LMQPSO only took about 15 iterations. Obviously, LMQPSO had the fastest convergence speed and was significantly better than other algorithms.

### 6.3. Simulation 1

**Figure 7:**
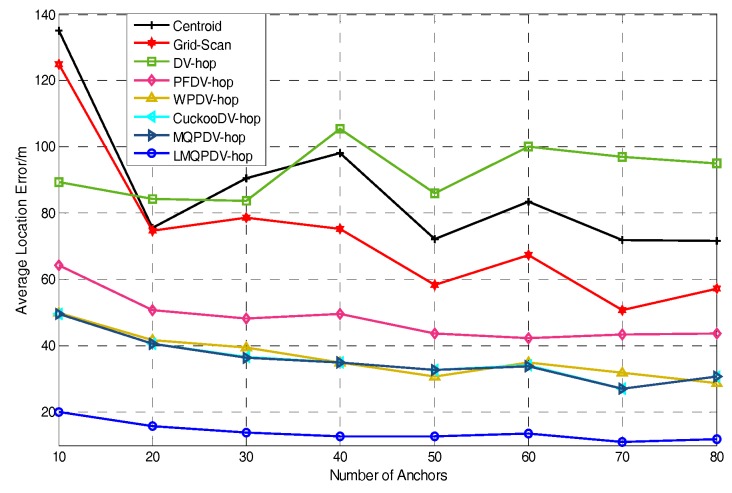
The effect of the number of anchors on error for WSN#1.

**Figure 8:**
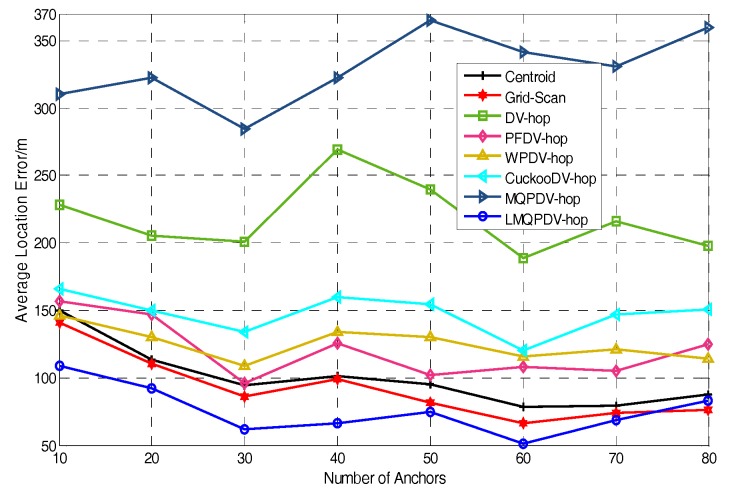
The effect of the number of anchors location on location error for WSN#2.

**Figure 9:**
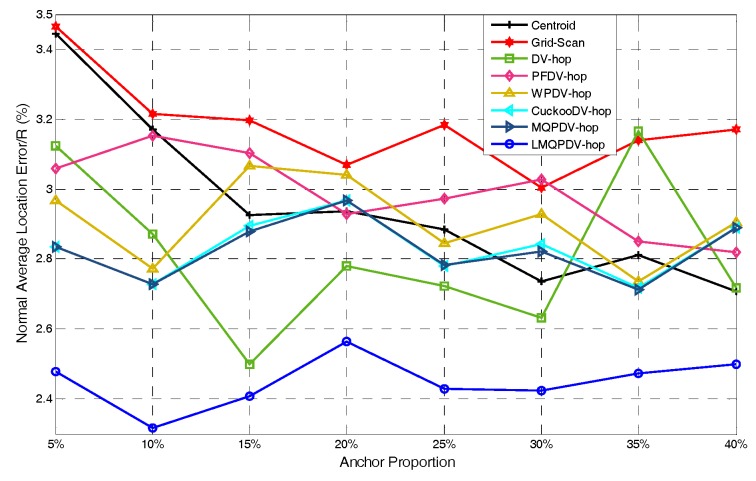
Communication range 150 for WSN#1.

**Figure 10:**
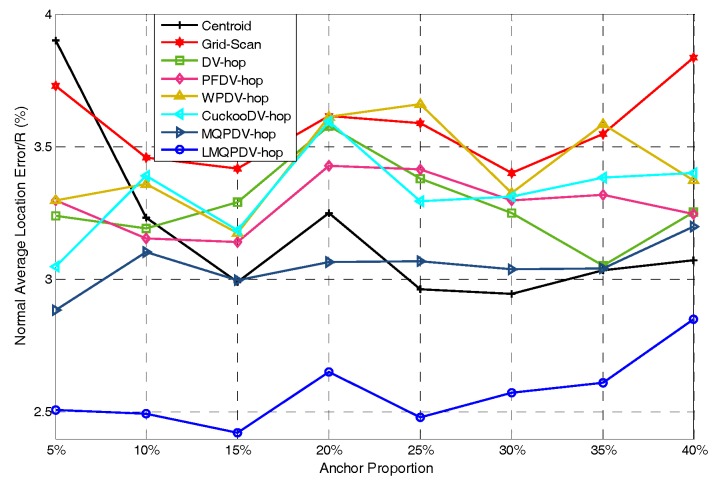
Communication range 150 for WSN#2.

**Figure 11:**
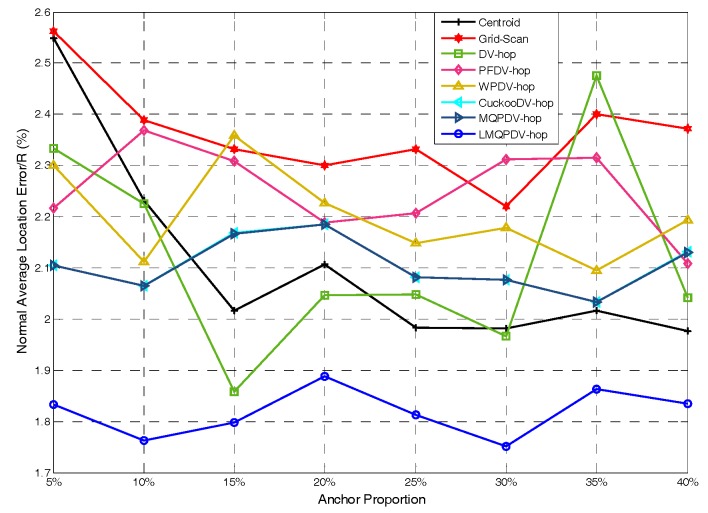
Communication range 200for WSN#1.

**Figure 12:**
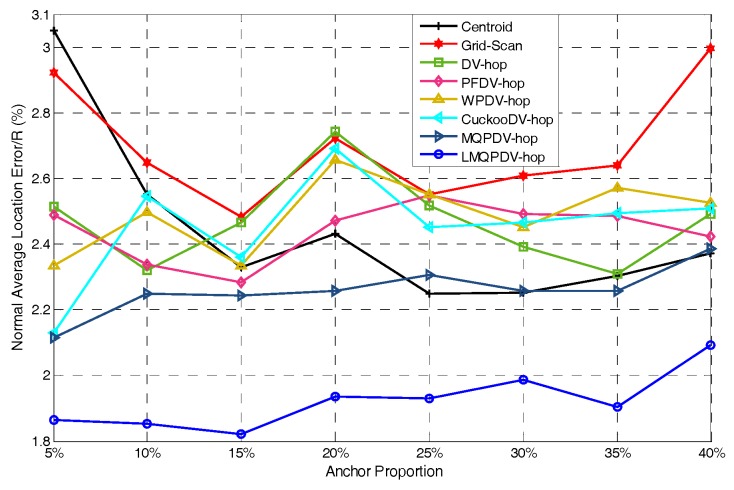
Communication range 200 for WSN#2.

**Figure 13:**
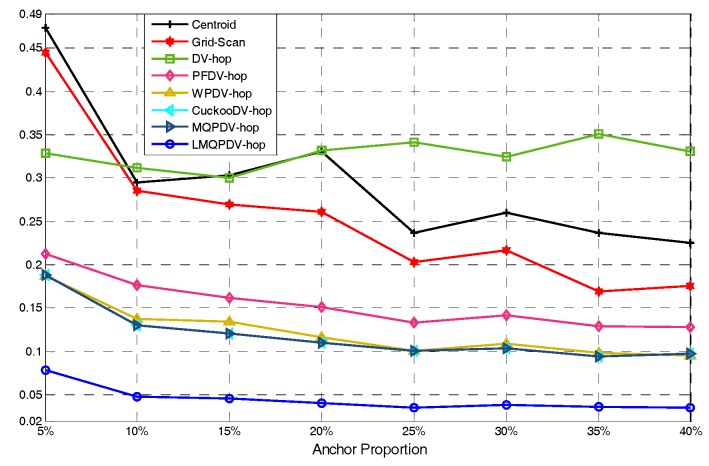
Communication range 250 for WSN#1.

**Figure 14:**
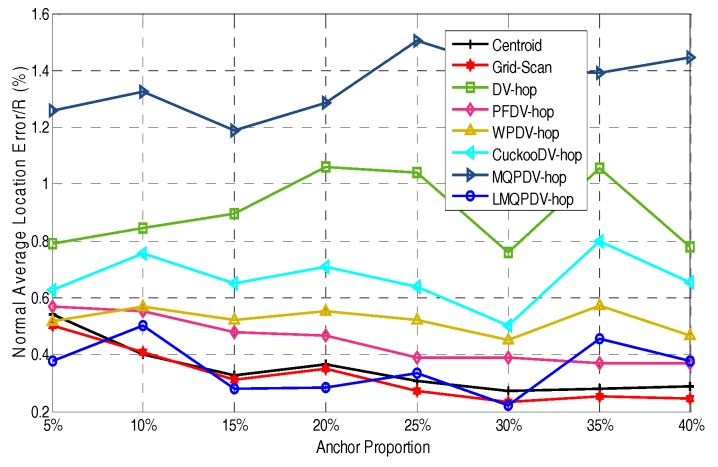
Communication range 250 WSN#2.

**Figure 15:**
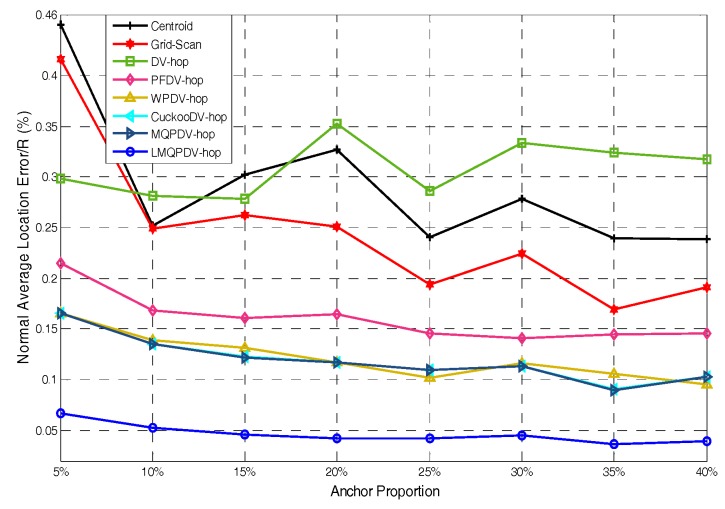
Communication range 300 for WSN#1.

**Figure 16:**
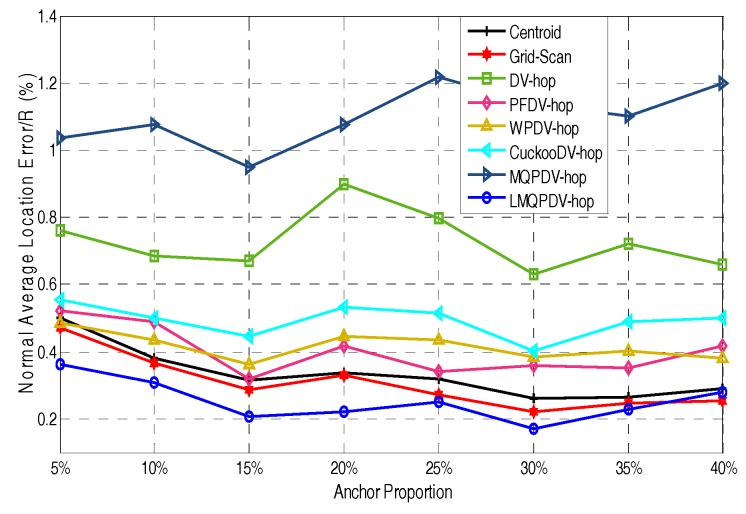
Communication range 300 WSN#2.

**Figure 17:**
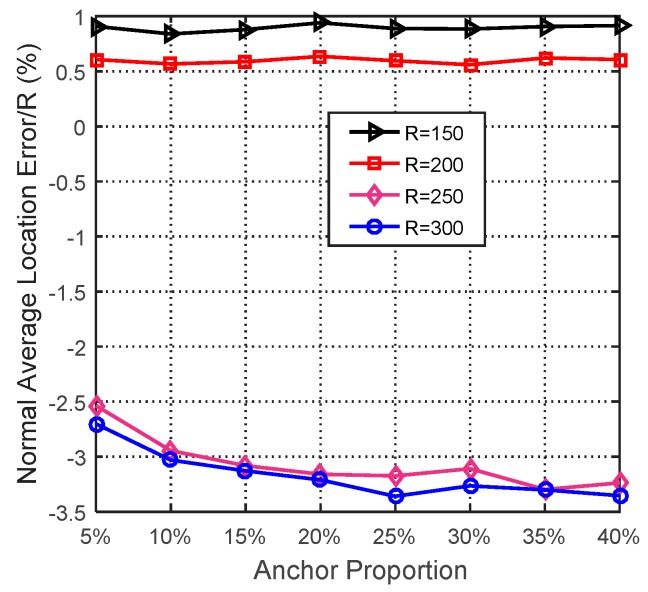
The influence of R for WSN#1.

**Figure 18:**
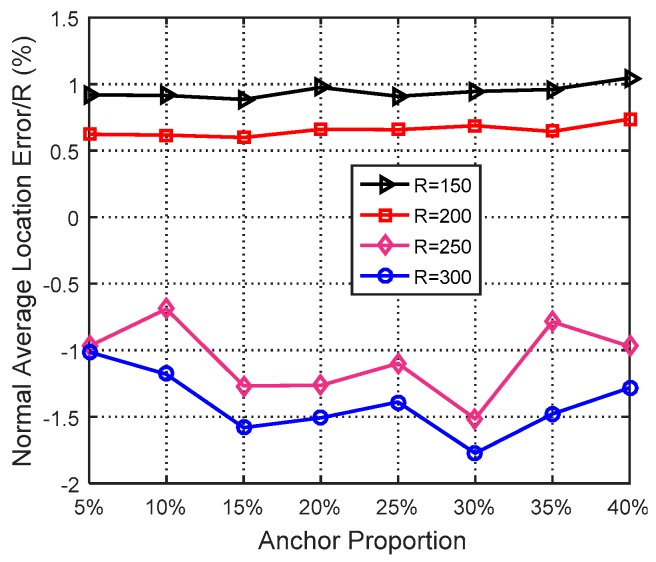
The influence of R for WSN#2.

**Figure 19:**
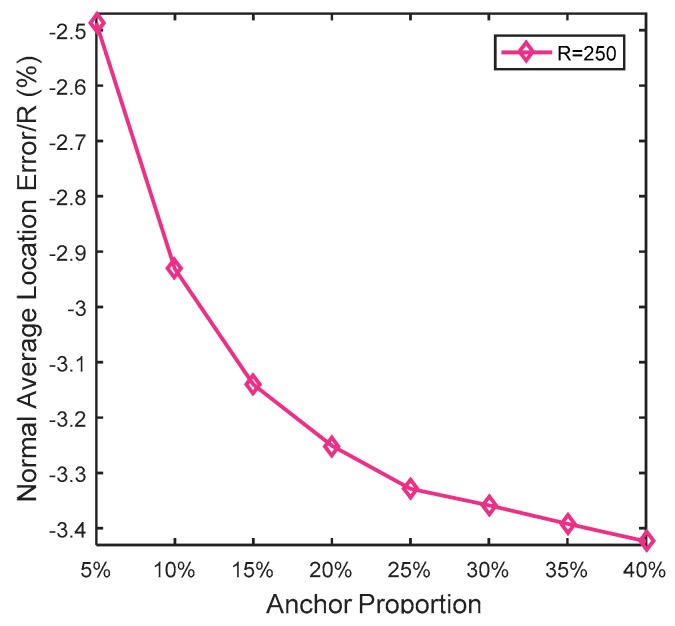
The influence of anchors for WSN#1.

**Figure 20:**
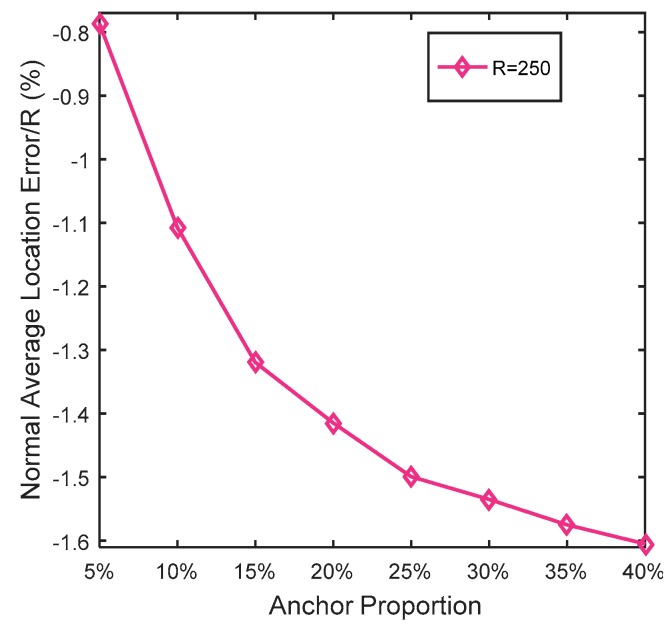
The influence of anchor WSN#2.

In this simulation, the number of anchor was set to the following values: 10, 20, 30, 20, 40, 50, 60, 70, 80. R was fixed to 300. Figure 7, Figure 8, Figure 9, Figure 10, Figure 11, Figure 12, Figure 13, Figure 14, Figure 15 and Figure 16 illustrate the effect of the number of anchors on the location error in eight localization schemes. These schemes, namely, LMQPDV-hop, MMQPDV-hop, CuckooDV-hop, PFDV-hop, WPDV-hop, DV-Hop Grid-scan and Centroid, were executed in WSN#1 and WSN#2. Figure 17, Figure 18, Figure 19 and Figure 20 illustrate the influences of the communication range and the number of anchor on the location error for only LMQPDV-hop. Table 3 and Table 4 give the average number of unresolved nodes (URN) for each localization scheme. In this case, a sensor node was unresolved sensor when its position could not be obtained using localization scheme.

For WNS#1, as shown in Figure 7, with the increase of the number of anchor, the location error curves of these schemes appear to gradually decline, except DV-hop and Centroid. The curves of DV-hop and Centroid appear to randomly fluctuate as the anchor number increased and the location error with the bigger number of anchors in these two curves was even larger than that with the smaller number. This is because the localization result of these two schemes depends heavily on the coordinates of the anchors rather than the number of anchors. When the coordinates of the anchors around an unknown node changed in these two schemes, the localization result of unknown node was changed immediately. However, in this simulation, a different number of anchors mean different random coordinates of anchors. In other words, the localization results of DV-hop and Centroid were changed at any time as the number of anchor changed. Moreover, a different number of anchors also mean different network topology. If some very unsatisfactory topologies are met unluckily by these two schemes, their location error maybe lead to an unreasonable phenomenon, that is, a larger number of anchors leads to a larger location error. Therefore, the curves in Figure 7 and Figure 8 appear to randomly jitter as the number of anchor increases. For the other localization schemes, the localization results depend not only on the coordinates of anchors, but also on the number of anchors. Thus, the curves in Figure 7 and Figure 8 show an overall decline along with the increasing number of anchors.

Among all these schemes, the location error of LMQPDV-hop was the smallest, and the fluctuation amplitude of its curve was also the smallest, which indicates that LMQPDV-hop had the best results and robustness.

For the irregular WSN scenario (WSN#2), the comparison results are shown in Figure 8. First, the location error of each scheme was significantly larger than that in WSN#1. Second, similar to WSN#1, the location error of LMQPDV-hop was still the smallest. Unlike WSN#1, the two non-SIA based methods (Grid-scan and Centroid) were ranked second and third. Unexpectedly, the location error of MMQPDV-hop was the largest. After more careful inspection, the non-SIA-based schemes had better localization accuracy than the SIA-based ones, except for LMQPDV-hop. This phenomenon can also be considered, as non-DV-hop-based schemes perform better than DV-hop-based ones, except for LMQPDV-hop. The main reason is that for DV-hop-based (or SIA-based) schemes, the C-shape non-deployment area created a huge error when calculating the average hop distance of the anchors. Here, the non-deployment area means that there was no communication between the two anchors in the area, i.e., the distance between the two anchors can be considered as infinite. The error of the average hop distance resulted in the error of the estimated distances calculated by Equation (24). Therefore, location error is generated when the estimated distance with the error is used to determinate the coordinate of an unknown node. In general, SIA-based schemes should have better localization result than least-squares-based one (used by DV-hop), but the MQPDV-hop is an exception. This is because MMQPSO, used by MQPDV-hop, prematurely fell into the local optimum during the process of optimization, and thus made its localization accuracy greater than DV-Hop. Besides, Centroid and Grid-Scan are anchor-intensive schemes, that is, the more anchors near the unknown node, the more accurate the localization results are.

For regular WSN#1, it can be seen from Figure 9, Figure 11, Figure 13 and Figure 15 that LMQPDV-hop achieved the highest localization accuracy with the same number of anchors regardless of the communication range. For the irregular WSN#2, it can be seen from Figure 10, Figure 12, Figure 14 and Figure 16 that LMQPDV-hop still kept the best performance except in the following two cases: R = 250 in Figure 14, and R = 300 and the anchor proportion = 40% in Figure 16.

Now we analyze the first case. As shown in Figure 14, LMQPDV-hop had better localization accuracy than other DV-Hop-based methods (including DV-Hop), whereas compared to the non-DV-Hop-based ones (i.e., Centroid and Grid-Scan), LMQPDV-hop achieved the highest accuracy in half of these eight scenarios (i.e., 5%, 15%, 20%, and 30%). At the same time, we also made a horizontal comparison with WSN #1 in the same WSN scenario (i.e., Figure 13). The localization accuracy of LMQPDV-hop was significantly higher than that of the Centroid and Grid-Scan, which also means that the C-shape non-deployment area will greatly affect the optimization result of LMQPSO. Figure 14 illustrates that LMQPDV-hop still outperformed others in most cases.

WSN #2, in other words, our new optimization algorithm LMQPSO still worked well in WSN#2. At the same time, we also noticed a detail in Figure 14. In the scenarios where the localization accuracy of LMQPDV-hop was not dominant, the location errors of other DV-hop-based schemes also showed similar deviations trends for the LMQPDV-hop. The reason is that the topology was randomly generated during deployment, and the non-uniform deployment of anchors (e.g., far away from the C-shape non-deployment area) made the average distance hop larger, thereby affecting the optimization of localization results.

For the second case, the localization accuracy of our scheme was only not the highest when the anchor proportion was 40% (it was still the second highest). The most likely reason is that the LMQPSO in our scheme rarely fell into the local optimum and could not jump out, which means that our scheme could not find the best localization results.

Table 3 and Table 4 show the scalability of all eight schemes in WSN#1 and WSN#2. As can be seen from Table 3 and Table 4, DV_hop-based schemes could find the coordinates of all the nodes in any case, regardless of their localization accuracy. But for non-DV-hop-based schemes (i.e., Centroid and Grid-scan), there were a certain number of unresolved nodes with small anchor proportions and communication ranges. Coincidentally, Centroid and Grid-scan had the same value of unresolved nodes, which indicates that these two schemes were essentially similar. Both Table 3 and Table 4 show that the number of URN decreased with the increase of anchor proportion and R, and the number of URN in WSN#2 was more serious than that in WSN#1.

More specifically, for WSN#1, when the anchor proportion ≤10% and R ≤ 250, Centroid and Grid-scan not only obtained the worst localization results, but also kept URNs. Obviously, an anchor proportion = 5% is better than an anchor proportion = 10%.

In contrast with WSN#1, for WSN#2, when anchor proportion were 5% and 10%, URNs existed in all the R values. Unlike WSN#1, even when anchor proportions increased to 15% and 20%, there were still URNs with R = 150. Notably, the number of URN with anchor proportion = 5% and R = 150 (i.e., 101) was almost half of all the nodes (i.e., 200). The main reason is that these URN could not find enough reference anchors because of the existence of the C-shap non-deployment area, especially for small number of anchor and R.

Now, let us study the influences of the following two factors, namely the communication rang and the number of anchors, on the localization result of LMQPDV-hop.

Figure 17 and Figure 18 show the influence of the communication rang. It is important to note that for the same communication rang in WSN#1 and WSN#2, the location errors with different anchor proportions can not be used to compare, because they were not obtained in the same network topology.

For WSN#1, we can see from Figure 17 that with the same anchor proportion, the longer the communication range is, the smaller the location error is. This is because a longer communication range means that an unknown node can find more anchors around it, which can improve the localization accuracy obviously.

For WSN#2, Figure 18 also illustrates the same conclusion as Figure 17, that is, that a longer communication range means a smaller location error. The main difference between them is that the location error in the regular WSN (i.e., WSN#1) was smaller than that in the irregular WSN (i.e., WSN#2), especially for R > 200. This is because the C-shape non-deployment area affected the average hop distance of anchor, which in turn affected the LMQPSO to find the optimized result.

Additionally, it should be noted that, as can be seen from Figure 17 and Figure 18, when the communication range of the node extended from 200 to 250, the localization accuracy was sharply improved. The reason is that when the communication range of nodes reaches or even exceeds 25% of the length of the sense target area, an unknown nodes can find enough anchors around it, which means a small error in the average hop distance and fewer hop counts. Both these factors can help the unknown node reduce the error in its estimated distances to anchors. And this, in turn, helped our localization scheme reduce the location error during our new LMQPSO optimization process. In addition, the location error with R = 300 seems to be only slightly smaller than R = 250 in WSN#1. The reason is that the number of anchors found by an unknown node may be sufficient when R = 250. However, Figure 18 shows that in WSN#2, R = 300 had a larger advantage than R = 250. This is because the C-shape non-deployment area enhanced the effectiveness of the communication range as the communication range increased from 250 to 300.

Figure 19 and Figure 20 show the influence of the number of anchors. Unlike Figure 17 and Figure 18, here, the anchors of WSN#1 and WSN#2 were chosen as follows: First, all the nodes were only randomly deployed once in WSN#1 and WSN#2. After deployment, each node is static and own its actual coordinate. Then, a certain number of nodes were randomly selected as anchors, and the rests acted as unknown nodes. In this way, the WSN topology of the first deployment remained the same throughout the selection process. All the anchor proportions in Figure 19 and Figure 20 were chosen randomly from the same WSN topology. Therefore, the location results for these different anchor proportions can be compared. As Figure 19 and Figure 20 show, the location errors in both WSN#1 and WSN#2 were decline as the anchor proportion increased, especially when the anchor proportion increased from 5% to 25%, the location error decreased sharply. This is because more anchors means a higher accuracy of the average hop distance and a fewer hop count, which can significantly reduce the error of the estimated distance from the unknown node to the anchors. Then, when these more accurate estimated distances were used to determinate the coordinates of the unknown nods, the location error could be reduced to improve the positioning accuracy.

At the same time, it also can be observed that the location errors in WSN#1 were smaller than that in WNS#2. The reason for this is that the C-shap non-deployment area affected the localization result.

### 6.4. Simulation 2

**Figure 21:**
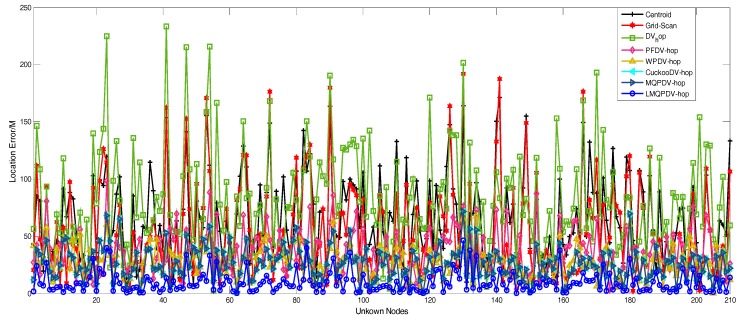
The location error of LMQPDV-hop when the anchor proportion was 30% and R = 250 for WSN #1.

**Figure 22:**
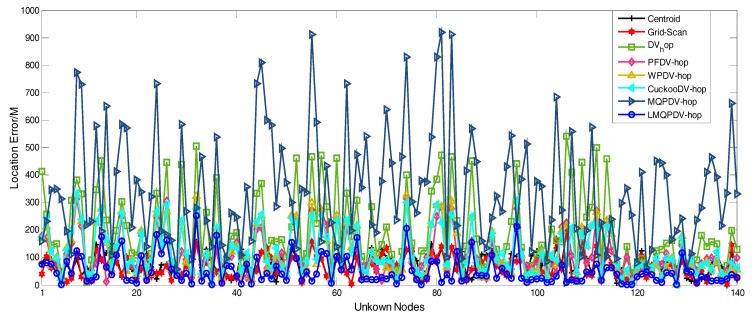
The location error of LMQPDV-hop when the anchor proportion was 30% and R = 250 for WSN#2.

Figure 21 and Figure 22 describe the average location error of each unknown node in all the localization schemes. Here, the anchor proportion is 30% and R = 250. The abscissa represents each unknown node, and the ordinate represents the average location error. As shown in Figure 21 and Figure 22, it seems that the average location error of LMQPDV-hop was the smallest.

Table 5 and Table 6 provide the statistical results of the location errors in WSN#1 and WSN#2, respectively. The best values in Table 5 and Table 6 are marked in bold. Table 5 shows that the SIAs-based DV-Hop variants outperformed the others (i.e., Centroid, Grid-Scan and DV-Hop) in the regular deployment scenario. By further analysis, LMQPDV-hop achieved the smallest Mean, SD, Median, Max and Min, which is to say that LMQPDV-hop achieved the best position accuracy (Mean = 9.5525 and Median = 6.9295) and had the best robustness in WSN#1. Table 6 shows that for WSN#2, LMQPDV-hop still achieved the best Mean and Median values, which indicates that LMQPDV-hop outperformed the other schemes.

However, unlike in Table 5, the location errors in the SIAs-based schemes were larger than in the Centroid and Grid-Scan, except for LMQPDV-hop. This is because the irregular (C-Sharp) deployment made the error of the average hop distance, which affected the SIAs to find the global optimum solution. It is worth noting that even in these unfriendly situations, our scheme can still achieve the optimal position (Mean = 50.9031 and Median = 35.4432).

In summary, it can be deduced that LMQPDV-hop is the best among all the schemes according to position accuracy.

## 7. Conclusions

In this paper, we proposed a new centralized range-free static node localization algorithm with higher accuracy (LMQPDV-hop) for WSNs. In LMQPDV-hop, a new optimization algorithm (LMQPSO) was designed to approach global optimal localization results rapidly by reconstructing the core components of original QPSO with MA and Lévy flight, and a comparative study was carried out. The experimental results show that the new LMQPSO performed better in terms of searching global optimization ability and convergence rate. Next, the distance estimation method of DV-hop was improved by the definition of the weight factor. Then, the more accurate estimated distances were optimized by a new LMPSO to determine the coordinates of unknown nodes. Extensive experiments have been performed in different WSN deployment scenarios to study the impacts of several factors, such as anchor density and communication range, on the proposed localization algorithm with respect to normalized average location error and localization success ratio. The simulation results verified the effectiveness of the new localization algorithm.

## Figures and Tables

**Table 1:** Simulation Parameters.

Parameter	Values	Parameter	Values
WSN#1: shape area (L × L) WSN#2: C shape area	$1000*1000\;m^{2}$ [1000 300 300 700]	Communication Rang R	150 m/200 m/250 m/300 m
Nodes N	300 for Area1; 200 for Area2	runs	300
Anchors M	Area1:15/30/45/60/75/90/105/120	$N_{p}$, $N_{nest}$	40
	Area2:10/20/30/40/50/60/70/80	Max Iteration	500

**Table 2:** Comparison results of five optimization algorithms on four benchmark functions at dimension 30 through 10 independent runs.

F	PSO1		PSO2		CS		MMQPSO		LMQPSO	
	Mean	SD	Mean	SD	Mean	SD	Mean	SD	Mean	SD
$f_{1}$	1.65 × 10^−15^	2.21 × 10^−15^	904.0898	506.6032	2.0397 × 10^−16^	8.11 × 10^−17^	3.39 × 10^−213^	6.76 × 10^−210^	**0**	**0**
$f_{2}$	56.787	23.1209	25.2722	3.8452	52.2067	5.5055	**0**	**0**	**0**	**0**
$f_{3}$	2018.1768	4.22 × 10^3^	4.22 × 10^−5^	1.14 × 10^−5^	**15.2697**	**2.0975**	28.7063	0.011424	27.2872	0.28203
$f_{4}$	0.011074	0.01344	1.8341	2.0568	2.81 × 10^−8^	8.42 × 10^−8^	**0**	**0**	**0**	**0**

**Table 3:** Number of unresolved sensors for WSN#1.

		Centroid	Grid-Scan	DV-hop	PFDV-hop	WPDV-hop	CuckooDV-hop	MMQPDV-hop	LMQPDV-hop
Anchors = 5%	R = 150	**39**	**39**	0	0	0	0	0	0
	R = 200	**17**	**17**	0	0	0	0	0	0
	R = 250	**2**	**2**	0	0	0	0	0	0
	R = 300	0	0	0	0	0	0	0	0
Anchors = 10%	R = 150	**10**	**10**	0	0	0	0	0	0
	R = 200	**6**	**6**	0	0	0	0	0	0
	R = 250	**2**	**2**	0	0	0	0	0	0
	R = 300	0	0	0	0	0	0	0	0
Anchors = 15%	R = 150	0	0	0	0	0	0	0	0
	R = 200	0	0	0	0	0	0	0	0
	R = 250	0	0	0	0	0	0	0	0
	R = 300	0	0	0	0	0	0	0	0
Anchors = 20%	R = 150	0	0	0	0	0	0	0	0
	R = 200	0	0	0	0	0	0	0	0
	R = 250	0	0	0	0	0	0	0	0
	R = 300	0	0	0	0	0	0	0	0
Anchors = 25%	R = 150	0	0	0	0	0	0	0	0
	R = 200	0	0	0	0	0	0	0	0
	R = 250	0	0	0	0	0	0	0	0
	R = 300	0	0	0	0	0	0	0	0
Anchors = 30%	R = 150	0	0	0	0	0	0	0	0
	R = 200	0	0	0	0	0	0	0	0
	R = 250	0	0	0	0	0	0	0	0
	R = 300	0	0	0	0	0	0	0	0
Anchors = 35%	R = 150	0	0	0	0	0	0	0	0
	R = 200	0	0	0	0	0	0	0	0
	R = 250	0	0	0	0	0	0	0	0
	R = 300	0	0	0	0	0	0	0	0
Anchors = 40%	R = 150	0	0	0	0	0	0	0	0
	R = 200	0	0	0	0	0	0	0	0
	R = 250	0	0	0	0	0	0	0	0
	R = 300	0	0	0	0	0	0	0	0

**Table 4:** Number of unresolved sensors for WSN#2.

		Centroid	Grid-Scan	DV-hop	PFDV-hop	WPDV-hop	CuckooDV-hop	MMQPDV-hop	LMQPDV-hop
Anchors = 5%	R = 150	**101**	**101**	0	0	0	0	0	0
	R = 200	**51**	**51**	0	0	0	0	0	0
	R = 250	**47**	**47**	0	0	0	0	0	0
	R = 300	**37**	**37**	0	0	0	0	0	0
Anchors = 10%	R = 150	**23**	**23**	0	0	0	0	0	0
	R = 200	**17**	**17**	0	0	0	0	0	0
	R = 250	**8**	**8**	0	0	0	0	0	0
	R = 300	2	2	0	0	0	0	0	0
Anchors = 15%	R = 150	**14**	**14**	0	0	0	0	0	0
	R = 200	**5**	**5**	0	0	0	0	0	0
	R = 250	**3**	**3**	0	0	0	0	0	0
	R = 300			0	0	0	0	0	0
Anchors = 20%	R = 150	**10**	**10**	0	0	0	0	0	0
	R = 200	0	0	0	0	0	0	0	0
	R = 250	0	0	0	0	0	0	0	0
	R = 300	0	0	0	0	0	0	0	0
Anchors = 25%	R = 150	**7**	**7**	0	0	0	0	0	0
	R = 200	0	0	0	0	0	0	0	0
	R = 250	0	0	0	0	0	0	0	0
	R = 300	0	0	0	0	0	0	0	0
Anchors = 30%	R = 150	0	0	0	0	0	0	0	0
	R = 200	0	0	0	0	0	0	0	0
	R = 250	0	0	0	0	0	0	0	0
	R = 300	0	0	0	0	0	0	0	0
Anchors = 35%	R = 150	0	0	0	0	0	0	0	0
	R = 200	0	0	0	0	0	0	0	0
	R = 250	0	0	0	0	0	0	0	0
	R = 300	0	0	0	0	0	0	0	0
Anchors = 40%	R = 150	0	0	0	0	0	0	0	0
	R = 200	0	0	0	0	0	0	0	0
	R = 250	0	0	0	0	0	0	0	0
	R = 300	0	0	0	0	0	0	0	0

**Table 5:** Comparison results of location error WSN#1 through 10 independent runs.

Algorithms	Mean (m)	SD	Median	Max	Min
Centroid	64.9569	38.3392	56.7564	171.5318	4.3869
Grid-Scan	54.1673	41.7036	41.2522	192.1353	2.3492
DV-Hop	81.0914	44.9711	74.1953	233.7833	2.0677
PSOPF	35.4215	19.0896	31.5747	98.6248	2.1356
WPDV-hop	27.3898	13.3948	26.4115	67.3779	0.4255
CuckooDV-hop	25.9650	14.1097	24.4645	71.5169	1.0470
MMQPDV-hop	25.9654	14.1100	24.4645	71.5157	1.0470
LMQPDV-hop	**9.5525**	**9.1495**	**6.9295**	**46.3154**	**0.2252**

**Table 6:** Comparison results of location error for WSN#2 through 10 independent runs.

Algorithms	Mean (m)	SD	Median	Max	Min
Centroid	78.2976	**40.0063**	73.9095	**187.5722**	5.1489
Grid-Scan	66.1192	41.0146	58.0107	192.2525	1.2345
DV-Hop	188.7333	138.1515	146.0290	541.8573	8.8043
PSOPF	107.7841	77.1498	82.7475	314.4339	12.7221
WPDV-hop	115.5668	89.4502	76.7918	328.2392	2.5448
CuckooDV-hop	120.2391	82.393	91.0585	335.0295	2.4837
MMQPDV-hop	341.6534	208.1381	300.2621	918.1644	8.0499
LMQPDV-hop	**50.9031**	50.1564	**35.4432**	251.2712	**0.1775**
